# Lysis of Endogenously Infected CD4+ T Cell Blasts by rIL-2 Activated Autologous Natural Killer Cells from HIV-Infected Viremic Individuals

**DOI:** 10.1371/journal.ppat.1000101

**Published:** 2008-07-11

**Authors:** Manuela Fogli, Domenico Mavilio, Enrico Brunetta, Stefania Varchetta, Khaled Ata, Gregg Roby, Colin Kovacs, Dean Follmann, Daniela Pende, Jeffrey Ward, Edward Barker, Emanuela Marcenaro, Alessandro Moretta, Anthony S. Fauci

**Affiliations:** 1 Laboratory of Immunoregulation, National Institute of Allergy and Infectious Diseases, National Institutes of Health, Bethesda, Maryland, United States of America; 2 Laboratory of Experimental Immunology, Istituto Clinico Humanitas, Rozzano, Milano, Italy; 3 Centre for Hepatology and Infectious Diseases, Policlinico San Matteo and University of Pavia, Italy; 4 Department of Medicine, University of Toronto, Toronto, Ontario, Canada; 5 Biostatistics Research Branch, National Institute of Allergy and Infectious Diseases, National Institutes of Health, Bethesda, Maryland, United States of America; 6 Istituto Nazionale per la Ricerca sul Cancro, Genova, Italy; 7 Department of Microbiology and Immunology, State University of New York, Upstate Medical University, Syracuse, New York, United States of America; 8 Department of Immunology and Microbiology, Rush University Medical Center, Chicago, Illinois, United States of America; 9 Dipartimento di Medicina Sperimentale, University of Genova, Genova, Italy; National Institutes of Health-NIAID, United States of America

## Abstract

Understanding the cellular mechanisms that ensure an appropriate innate immune response against viral pathogens is an important challenge of biomedical research. *In vitro* studies have shown that natural killer (NK) cells purified from healthy donors can kill heterologous cell lines or autologous CD4+ T cell blasts exogenously infected with several strains of HIV-1. However, it is not known whether the deleterious effects of high HIV-1 viremia interferes with the NK cell-mediated cytolysis of autologous, endogenously HIV-1-infected CD4+ T cells. Here, we stimulate primary CD4+ T cells, purified *ex vivo* from HIV-1-infected viremic patients, with PHA and rIL2 (with or without rIL-7). This experimental procedure allows for the significant expansion and isolation of endogenously infected CD4+ T cell blasts detected by intracellular staining of p24 HIV-1 core antigen. We show that, subsequent to the selective down-modulation of MHC class-I (MHC-I) molecules, HIV-1-infected p24^pos^ blasts become partially susceptible to lysis by rIL-2-activated NK cells, while uninfected p24^neg^ blasts are spared from killing. This NK cell-mediated killing occurs mainly through the NKG2D activation pathway. However, the degree of NK cell cytolytic activity against autologous, endogenously HIV-1-infected CD4+ T cell blasts that down-modulate HLA-A and –B alleles and against heterologous MHC-I^neg^ cell lines is particularly low. This phenomenon is associated with the defective surface expression and engagement of natural cytotoxicity receptors (NCRs) and with the high frequency of the anergic CD56^neg^/CD16^pos^ subsets of highly dysfunctional NK cells from HIV-1-infected viremic patients. Collectively, our data demonstrate that the chronic viral replication of HIV-1 in infected individuals results in several phenotypic and functional aberrancies that interfere with the NK cell-mediated killing of autologous p24^pos^ blasts derived from primary T cells.

## Author Summary

Natural killer (NK) cells represent an important line of defense against viral infections. *In vitro* studies with exogenously infected CD4+ T cell blasts from healthy donors have demonstrated that NK cells can kill autologous HIV-1-infected target cells. However, the ability of NK cells from HIV-1-infected viremic patients to kill autologous, endogenously infected CD4+ T cells had never been examined and remains uncertain. Given the reported abnormalities in phenotype and functions of NK cells from HIV-infected viremic individuals, we determined the function of NK cells in killing HIV-1-infected target cells under conditions that more closely mimic the *in vivo* environment in HIV-infected individuals. We show that NK cells from HIV-1-infected viremic patients display a variable although generally low ability to selectively eliminate autologous and endogenously HIV-1-infected CD4+ T cell blasts expanded *ex vivo* from peripheral blood. Various factors, including the markedly defective engagement of important NK cell activation pathways and high frequencies of the pathologic CD56^neg^/CD16^pos^ NK cell subset in HIV-1-infected viremic patients, influenced NK cell–mediated cytolysis of endogenously infected CD4+ T cell blasts.

## Introduction

Natural killer (NK) cells are important effectors of innate immune responses and are capable of providing cellular immunity against tumor-transformed and virally-infected cells, without prior antigen sensitization [1],[2]. Among the several NK cell effector-functions, spontaneous killing of non-self targets was the first to be described and is the reason they were named “natural killer” cells [3]. NK cell cytolytic machinery is modulated by a delicate balance between opposing signals delivered by two heterogeneous families of inhibitory and activating NK cell receptors. Under physiological conditions, cytotoxicity against normal autologous cells is blocked by the specific recognition of different MHC class –I (MHC-I) molecules by inhibitory NK cell receptors (iNKRs). Interactions between iNKRs and MHC-I, tolerance to self, and determination of the extent of cytolytic activity are achieved through a complex process that educates NK cells to ensure self-recognition [4]. Diminution or absence of expression of HLA-I alleles on a cell surface following viral infection or tumor transformation results in the reduced engagement of iNKRs and allows activating NK receptors and co-receptors to trigger NK cell-mediated cytolysis [5].

Several studies have already described numerous aberrancies of NK cell phenotype and function in chronically HIV-1 infected patients with high levels of ongoing viral replication. These abnormalities include aberrant expression and function of several iNKRs and natural cytotoxicity receptors (NCRs), markedly impaired cytolytic activity against tumor cell targets, defective production of important antiviral cytokines [6],[7] and defective interactions with autologous dendritic cells (DCs) [8]. All of these phenotypic and functional perturbations are particularly pronounced in an unusual CD56^neg^/CD16^pos^ (CD56^neg^) NK cell subset that is preferentially expanded in HIV-1 infected viremic patients [9],[10],[11].

Because the frequency of peripheral blood CD4+ T cells that harbor replication-competent virus is extremely low in HIV-1 infected patients [12],[13], it remains to be determined whether highly dysfunctional NK cells from patients with high levels of ongoing viral replication are able to eliminate autologous and endogenously HIV-1 infected CD4+ T cells.

In order to further understand the direct effects of HIV-1 on CD4+ T cells and other cell types, several models of *in vitro* infection with different HIV-1 strains have been developed. Through these experimental methods, several reports show that HIV-1 selectively down-modulates HLA-A and -B alleles in both cell lines and CD4+ T cell-derived blasts [14],[15],[16]. It has been also demonstrated that the ability of NK cells from healthy donors to kill autologous and exogenously infected CD4+ T cell blasts is influenced by modulation by the exogenous virus of ligands for inhibitory and activating NK cell receptors on these primary T cell blasts [15],[17]. Even though these approaches using *in vitro* infection have significantly contributed to understanding the cellular interactions between NK cells and autologous HIV-1 infected CD4+ T cell blasts, it is still unclear what role, if any, NK cells obtained from HIV-1 infected viremic patients play in the clearance of endogenously infected autologous CD4+ T cells *ex vivo*.

In the present study, we describe the killing of endogenously infected CD4+ T cell blasts by autologous NK cells from HIV-1 infected viremic individuals. In addition, we describe several mechanisms involved in the regulation of this NK cell-mediated killing. Finally, we characterize phenotypically the endogenously HIV-1 infected CD4+ T cell blasts used as targets in this experimental system.

## Results

### Establishment of CD4+ T cell blasts endogenously infected with HIV-1

**Figure 1:**
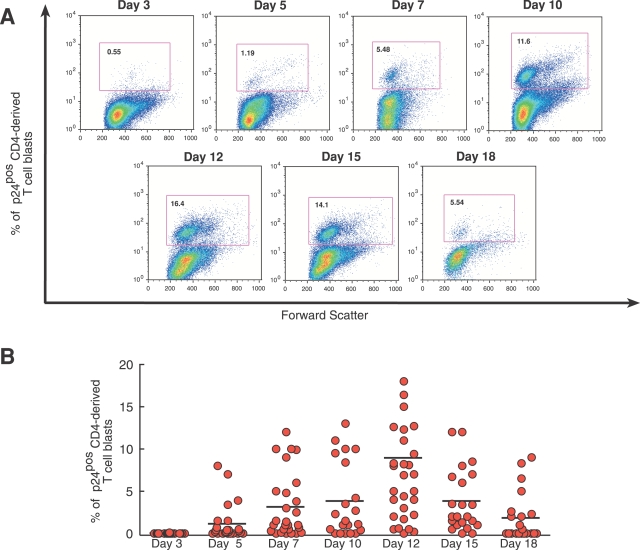
Establishment of endogenously HIV-1 infected CD4+ T cell blasts. (A) Percentages of p24^pos^ blasts (open squares) expanded after activation with PHA and rIL-2 in a time-course experiment from a representative HIV-1 infected viremic patient. (B) Summary graph of statistical dot plots showing the percentages with medians (horizontal black bars) of p24^pos^ blasts expanded over time after stimulation with PHA and rIL-2 from a cohort of infected viremic patients.

It has been reported previously that activation *in vitro* with different stimuli of either total PBMCs or purified CD4+ T cells from HIV-1 infected individuals induces viral expression and replication [18],[19],[20],[21]. The number of HIV-1 virions produced by these endogenously infected activated blasts was detected through real-time PCR, while the amount of p24 HIV-1 core antigen released in culture supernatant was determined by ELISA. Using an experimental approach similar to that for activating primary T cells *in vitro* (Figure S1), we sought to isolate and characterize these productively infected cells starting from highly enriched CD4+ T cells purified from HIV-1 infected viremic patients. As shown in Figure 1A, 5–7 days of stimulation with phytohemoagglutinin (PHA) and recombinant IL-2 (rIL2) were required to observe a variable but consistent percentage of endogenously HIV-1 infected CD4+ T cell blasts, characterized by the presence of intracellular viral p24 core antigen. In order to determine the peak of maximal expansion of these infected cells, we tested the amount of p24 antigen in CD4+ T cell blasts every 3 days for 3 weeks. Within our cohort of HIV-1 infected viremic donors, the highest percentages of p24^pos^ expression in activated CD4+ T cell blasts were detected, on average, after 12 days of activation (median: 9.43%; SD = ±6.6) and started to decrease progressively after this time point (Figure 1B).

Of the other several stimuli used to expand HIV-1 infected CD4+ T cell blasts, only PHA plus rIL-2 and rIL-7 achieved similar and sometimes better results after 12 days in culture compared to stimulation with PHA and rIL-2 (Figure S2).

### Physiological status and phenotype of p24^pos^ CD4+ T cell blasts

**Figure 2:**
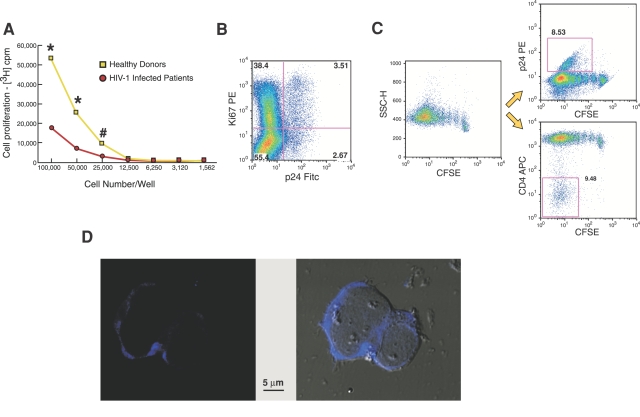
Physiological status of CD4+ T cell blasts at the day of maximal expansion. (A) Proliferation of total CD4+ T cell blasts obtained from uninfected and HIV-1 infected viremic individuals at different cell numbers in culture. Data are presented as median of experiments performed in triplicate on 5 healthy donors and 5 HIV-1 infected viremic individuals. (* p<0.0001; # p = 0.009). (B) Percentages of p24^neg^ (upper left quadrant of dot plot graph) and p24^pos^ (upper right quadrant of dot plot graph) blasts undergoing cell cycling (Ki67^pos^ cells) in a representative HIV-1 infected viremic patient. (C) Multicolor flow cytometric analysis showing the down-modulation of CD4 in a subset of p24^pos^ blasts (open squares) within proliferating CD4+ T cell blasts analyzed by CFSE dye dilution in a representative HIV-1 infected viremic patient. (D) Fluorescence microscopic image of two endogenously HIV-1 infected CD4+ T cell blasts. The HIV-1 p24 core antigen is stained blue within the cytoplasmic compartment of the cells.

**Figure 3:**
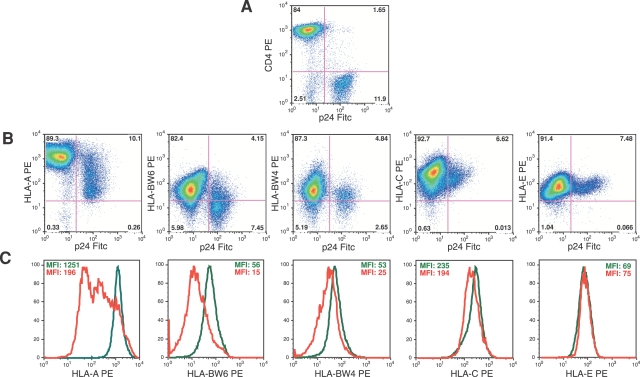
Phenotype of CD4+ T cell blasts. Surface expression of CD4, HLA-A, HLA-BW4, HLA-BW6, HLA-C and HLA-E by p24^neg^ (upper left quadrants of dot plot graphs and green lines in histogram graphs) and p24^pos^ (upper right quadrants of dot plot graphs and red lines in histogram graphs) blasts in a representative HIV-1 infected viremic patient. Data are presented as percentage of expression (A–B) and as geometric mean fluorescence intensity (MFI) (C).

We then analyzed whether p24^pos^ CD4+ T cell blasts were able to proliferate during the period of maximal expansion. As expected, the ability of unfractionated CD4+ T cell blasts from HIV-1 infected patients to undergo proliferation was significantly lower compared to that of unfractionated blasts from healthy donors (Figure 2A). Even though the positive expression of Ki67 nuclear antigen by p24^pos^ fractions of CD4+ T cell blasts indicated that these endogenously infected cells were able to enter the cell cycle (Figure 2B), it has been shown both *in vitro* and *ex vivo* that they are arrested in G2/M stage and do not complete the cell cycle [22],[23]. In order to correlate the kinetics of expansion of these endogenously infected blasts with cell proliferation and CD4 expression, we analyzed the dilution of the vital dye carboxyfluorescein diacetate succinimidyl ester (CFSE) in CD4+ T cell blasts using a multicolor flow cytometric approach. After 12 days of stimulation, a subset of proliferating CFSE-labeled blasts showed active intracellular viral replication (p24^pos^ cells) with a simultaneous down-modulation of cell surface CD4 (Figure 2C). Therefore, the loss of CD4 is associated with a productive infection in either endogenously (Figure 3A) or exogenously infected CD4+ T cell-derived blasts [17],[24],[25]. We also visualized the intracellular HIV-1 p24 core antigens in endogenously infected CD4+ T cell blasts by fluorescence microscopy (Figure 2D).

It has also been reported that HIV-1 infection *in vitro* results in a selective down-modulation of MHC-I molecules in cell lines and in exogenously infected primary CD4+ T cells [14],[15],[16]. In order to determine whether this phenomenon also occurs in endogenously infected CD4+ T cell blasts expanded from HIV-1 infected individuals, we analyzed the expression of classic and non-classic HLA molecules on p24^pos^ and p24^neg^ blasts. We found that surface levels of HLA-A and -B alleles, calculated as mean fluorescence intensity, were significantly down modulated on p24^pos^/CD4^neg^ blasts compared to p24^neg^/CD4^pos^ blasts (p = 0.004 for HLA-A alleles; p = 0.018 for HLA-BW4 and –BW6 alleles). In contrast, the expression of HLA-C and HLA-E molecules did not substantially differ between p24^neg^ and p24^neg^ blasts (Figures 3B–C and Table 1).

**Table 1:** Surface levels of HLA-I molecules on p24^pos^ and p24^neg^ T cell blasts

Patients	HLA-A		HLA-Bw4		HLA-Bw6		HLA-C		HLA-E	
	p24^pos^	p24^neg^	p24^pos^	p24^neg^	p24^pos^	p24^neg^	p24^pos^	p24^neg^	p24^pos^	p24^neg^
1	2017	***862***	70	***37***	neg	***neg***	147	126	44	39
2	2342	***1221***	115	***28***	24	***10***	51	53	87	78
3	1440	***428***	neg	***neg***	71	***28***	294	251	81	85
4	1080	***400***	neg	***neg***	44	***19***	45	32	8	12
5	2045	***870***	610	***418***	neg	***neg***	33	34	53	45
6	1251	***196***	56	***15***	53	***25***	235	194	69	77
7	685	***295***	115	***28***	24	***10***	201	195	24	43
8	625	***249***	54	***30***	18	***10***	42	53	72	75
9	869	***388***	64	***33***	48	***28***	69	75	82	85
10	607	***261***	neg	***neg***	neg	***neg***	134	124	41	54
Median	1166	***394***	56	***28***	44	***19***	101	99	61	64

Results are presented as mean fluorescence intensities (MFI) on cells from 10 representative HIV-1 infected viremic patients. Down-modulation of HLA-A and -B alleles is highlighted in bold italic.

### NK cell-mediated killing of autologous p24^pos^ and p24^neg^ T cell blasts

#### Role of MHC-I molecules

**Figure 4:**
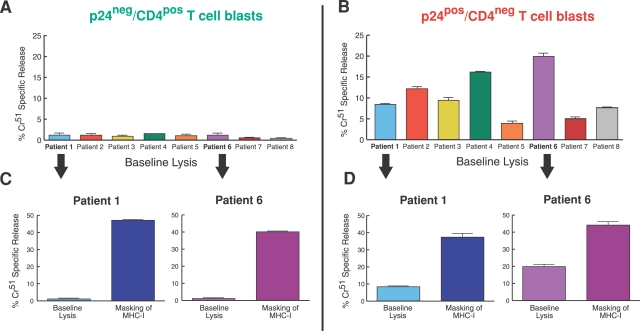
NK cell-mediated killing of autologous CD4+ T cell blasts: role of MHC-I molecules. Cytolysis of autologous p24^neg^/CD4^pos^ and p24^pos^/CD4^neg^ blasts by rIL-2 activated NK cells purified from HIV-1 infected viremic patients. Cells were incubated either in the absence (baseline lysis in A and B) or in the presence (C and D) of specific mAbs masking classic HLA-A/-B/-C and non classic HLA-E molecules. Data shown are from experiments performed in triplicate (±SD) using cells from 8 (A–B) and 2 (C–D) representative HIV-1 infected viremic individuals. The NK cell:CD4-derived blast ratio in all experiments was 10:1.

Cytolytic activity of NK cells against autologous cells is possible when the usually dominant inhibitory interaction between MHC-I molecules and iNKRs is either absent or weak. Although it has been previously reported that the percentage of NK cells positive for iNKRs is either maintained or increased in HIV-1 infected viremic patients [7], the selective down-modulation of HLA alleles on endogenously HIV-1 infected CD4+ T cell-derived blasts would, theoretically, make these cells sensitive to lysis exerted by autologous NK cells. In order to verify this issue, we separated infected from uninfected T cell blasts on the basis of CD4 surface expression (Figure S3), and we assessed the ability of rIL-2 activated autologous NK cells to kill both p24^neg^/CD4^pos^ and p24^pos^/CD4^neg^ blasts. As expected, p24^neg^/CD4^pos^ blasts with normal levels of MHC-I were highly resistant to NK cell-mediated cytolysis. In contrast, the percentages of infected p24^pos^/CD4^neg^ blasts lysed by autologous NK cells was significantly higher compared to that of p24^neg^/CD4^pos^ blasts (p = 0.003) (Figure 4A–B). To further determine the importance of the down-modulation of HLA-A and -B alleles in rendering infected blasts sensitive to NK cell lysis, we repeated the same experiment in the presence of specific anti-MHC-I monoclonal antibodies (mAbs). In fact, the complete blocking of interactions between iNKRs and HLA-I molecules would theoretically render both infected and uninfected CD4+ T cell blasts equally susceptible to NK cell-mediated killing. Indeed, the masking of all MHC-I molecules abolished the inhibitory effect of iNKRs and resulted in a remarkably higher and comparable NK cell-mediated lysis of both p24^neg^ and p24^pos^ blasts (Figure 4C–D).

Despite the significantly higher susceptibility of endogenously HIV-1 infected blasts, compared to uninfected blasts, to be lysed by autologous NK cells, the degree of NK cell cytolytic activity against p24^pos^/CD4^neg^ cell blasts was still relatively low and variable from patient to patient (range: 4.2%–23%; median: 9.6%). Even when interactions between iNKRs and MHC-I were completely blocked, NK cells only partially eliminated both p24^pos^ and p24^neg^ autologous CD4+ T cell blasts (range: 13%–48%; median: 38.4%) (Figure 4). In order to understand whether NK cells from HIV-1 infected viremic patients, compared to healthy donors, were defective in killing cells with low or no expression of MHC-I molecules, we analyzed the NK cell-mediated cytolysis of cell lines that do not express classic and non-classic HLA-I alleles. In line with data previously reported [6],[7], we confirmed that the ability of NK cells from HIV-1 infected viremic patients to lyse K562 and 221 cell lines was significantly lower compared with that of uninfected individuals (Figure S4). A possible explanation for this low cytolytic ability by NK cells from HIV-1 infected viremic individuals against both MHC-I^neg^ cell lines or HIV-1 endogenously infected CD4+ T cell blasts that down-modulate HLA-A and –B alleles might be the defective engagement of activating NK cell receptors.

#### Role of NCRs and CD56^neg^ NK cell subset

If the dominant and negative effect of interactions between iNKRs and MHC-I molecules is partially overcome by the HIV-1 induced selective down-modulation of HLA-A and –B alleles, NK cell killing of autologous and endogenously infected CD4+ T cell blasts must occur through activating NK cell receptors. In order to identify those NK receptor(s) that trigger this NK cell-mediated lysis, we analyzed the phenotype and functions of NCRs and NKG2D that, under physiological conditions, represent the major activating receptors that regulate NK cell cytotoxicity.

**Figure 5:**
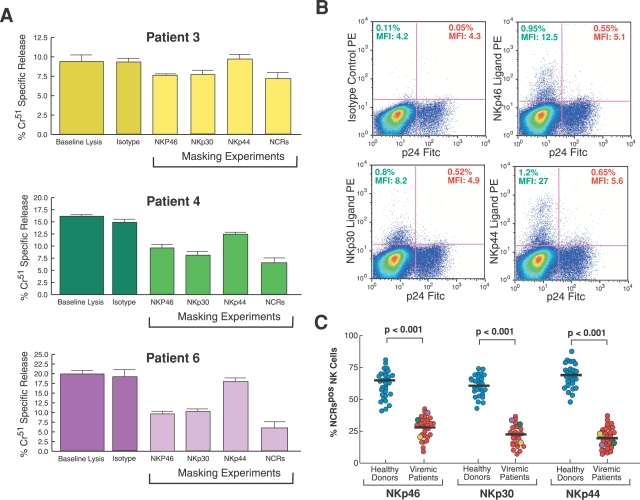
NK cell-mediated killing of autologous CD4+ T cell blasts: role of NCRs. (A) Cytolysis (in triplicate ±SD) of autologous p24^pos^/CD4^neg^ blasts by rIL-2 activated NK cells purified from HIV-1 infected viremic patients. Cells were incubated either in the absence (baseline lysis) or in the presence of specific mAbs masking NKp46, NKp30 and NKp44. Experiments were also performed masking the effector with anti NKp46, NKp30 and NKp44 (NCRs) simultaneously. Data shown are from experiments performed in triplicate (±SD) using cells from 3 representative HIV-1 infected viremic individuals. We used an anti-human CD56 IgM mAb as an isotype control for masking experiments. The NK cell:CD4-derived blast ratio in all experiments was 10∶1. (B) Surface expression of ligands for NKp44, NKp30 and NKp44 by p24^neg^ (upper left quadrants of dot plot graphs) and p24^pos^ (upper right quadrants of dot plot graphs) blasts derived from a representative HIV-1 infected viremic patient. The relative percentage of expression and geometric mean fluorescence intensity (MFI) for each ligand is indicated in the upper left (for p24^neg^ CD4-derived T cell blasts) and upper right (p24^pos^ CD4-derived T cell blasts) quadrants of each dot plot. (C) Summary graphs of statistical dot plots with medians (horizontal black bars) showing the percentages of total rIL-2 activated NKp46^pos^, NKp30^pos^, and NKp44^pos^ NK cells purified from healthy donors (blue circles) and HIV-1 infected viremic individual (red circles). Yellow, green and purple circles represent the three representative HIV-1 patients whose NK cell cytolytic functions are shown in Figures 4 and 5.

**Figure 6 ppat-1000101-g006:**
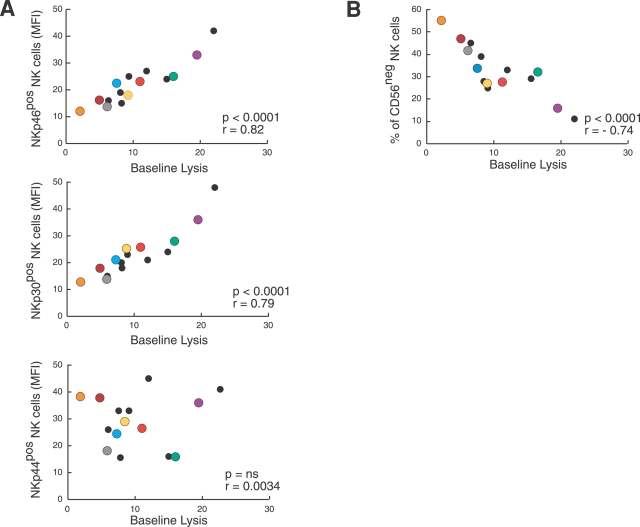
NCRs and CD56^neg^ cell impact on baseline lysis of endogenously infected CD4+ T cell blasts. Statistical analyses showing the correlation between the NK-cell mediated baseline lysis of autologous p24^neg^/CD4^pos^ blasts and the NK cell surface levels of NKp46, NKp30, NKp44 (MFI on IL-2 activated NK cells) (Panel A) and CD56 (percentages on freshly purified NK cells) (Panel B). Orange, brown, gray, blue, yellow, red, green and purple circles represent the eight representative HIV-1 patients whose NK cell cytolytic functions are also shown in Figure 4.

We performed masking experiments to address the role of NCRs in the NK cell lysis of autologous and endogenously HIV-1 infected CD4+ T cell blasts. The blocking of NKp46, NKp30 and NKp44 did not result in a significant decrease in NK cell-mediated lysis of p24^pos^/CD4^neg^ cell blasts, indicating that the NCRs were not playing a substantial role in this killing (Figure 5A). The lack of significant NK cell killing of endogenously HIV-1 infected CD4+ T cell blasts through the NCR activation pathway was also consistent with previously reported data showing that the percentage of NK cells expressing NCRs was significantly decreased on fresh and rIL-2 activated NK cells from HIV-1 viremic patients compared to those from healthy donors (Figure 5C) [6],[7]. Nevertheless, among those few HIV-1 infected viremic patients with higher levels of NKp46^pos^ and NKp30^pos^ NK cells, we could detect a more pronounced inhibition of NK cell-mediated killing especially after the simultaneous masking of three NCRs (Figure 5A; Patients 4 and 6). In this context, we found a highly significant correlation between the low degrees of NK cell-mediated lysis of p24^pos^/CD4^neg^ blasts and the decreased NK cell surface levels of NKp46 and NKp30, indicating that the low levels of expression of these two activating NK cell receptors negatively contribute to the killing of HIV-1 infected cells. Regardless of its expression on NK cells, NKp44 did not show any substantial contribution to NK cell cytolysis of p24^pos^/CD4^neg^ blasts (Figures 5A and 6A).

We also analyzed the expression of cellular ligands for NCRs using soluble human NCR-Ig fusion proteins. We found that the surface levels of NCR ligands on p24^pos^/CD4^neg^ cell blasts were very low, while small fractions of p24^neg^/CD4^pos^ cell blasts were positive for such ligands (Figure 5B). The fact that the ligands for NCRs were expressed at very low levels on naturally HIV-1 infected blasts did not correlate with the low degree of NK cell-mediated lysis of p24^pos^/CD4^neg^ cell blasts through NKp46, NKp30 or NKp44 pathways (data not shown). These data raise the question of whether soluble human Ig fusion proteins may be sensitive enough in detecting all ligands for NCRs.

We previously reported that among HIV-1 infected viremic individuals there is a high frequency of a markedly dysfunctional CD56^neg^ subset of NK cells in which surface expression of NKp46 and NKp30 and cytolytic activitiy against tumor cell line targets were found to be very low [11]. In this regard, we found that the degree of NK cell baseline cytolysis of autologous p24^pos^ blasts is inversely correlated with the frequencies of anergic CD56^neg^ NK cells (Figure 6B).

#### Role of NKG2D

**Figure 7:**
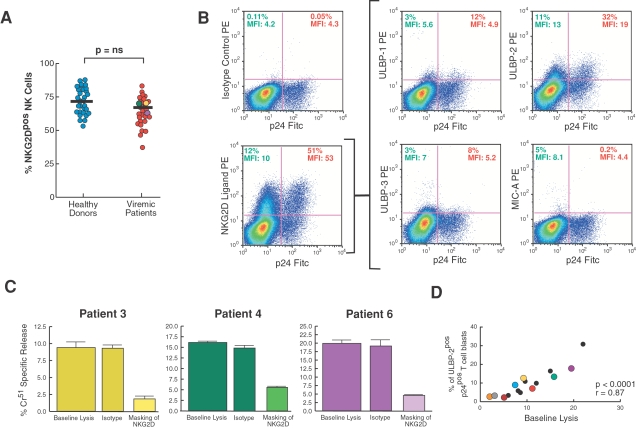
NK cell-mediated killing of autologous CD4+ T cell blasts: role of NKG2D. (A) Summary graphs of statistical dot plots with medians (horizontal black bars) showing the percentages of total rIL-2 activated NKG2D^pos^ NK cells purified from healthy donors (blue circles) and HIV-1 infected viremic individual (red circles). Yellow, green and purple circles represent the three representative HIV-1 patients whose NK cell cytolytic functions are shown in Figures 4 and 5. The NK cell:CD4-derived blast ratio in all experiments was 10∶1. (B) Surface expression of ligands for NKG2D by p24^neg^ (upper left quadrants of dot plot graphs) and p24^pos^ (upper right quadrants of dot plot graphs) blasts derived from a representative HIV-1 infected viremic patient. The relative percentage of expression and geometric mean fluorescence intensity (MFI) for each ligand is indicated in the upper left (for p24^neg^ CD4-derived T cell blasts) and upper right (p24^pos^ CD4-derived T cell blasts) quadrants of each dot plot. (C) Cytolysis (in triplicate ±SD) of autologous p24^pos^/CD4^neg^ blasts by rIL-2 activated NK cells purified from HIV-1 infected viremic patients. Cells were incubated either in the absence (baseline lysis) or in the presence of specific mAbs masking NKG2D. Data shown are from experiments performed in triplicate (±SD) using cells from 3 representative HIV-1 infected viremic individuals. We used an anti-human CD56 IgM mAb as an isotype control for masking experiments. The NK cell:CD4-derived blast ratio in all experiments was 10∶1. (D) Statistical analyses showing the correlation between the NK-cell mediated baseline lysis of autologous HIV-1 infected blasts and the percentages of ULBP-2 ligand for NKG2D on p24^pos^/CD4^neg^ cell blasts. Orange, brown, gray, blue, yellow, red, green and purple circles correspond to the eight representative HIV-1 patients whose NK cell cytolytic functions are also shown in Figures 4 and 6.

As we previously showed [7], there were no differences in NK cell surface levels of NKG2D between infected and uninfected individuals (Figure 7A). We also found that NKG2D ligands, detected through a soluble human NKG2D-Ig fusion protein, were expressed at high levels on endogenously HIV-1-infected CD4+ T cell-derived blasts. In particular, among the previously described NKG2D ligands [1], ULBP molecules (in particular ULBP-2) were clearly expressed on p24^pos^ blasts (Figure 7B), while the levels of MIC -A (Figure 7B) and –B (data not shown) were almost undetectable.

As previously demonstrated for NCRs, we then performed masking experiments to understand the extent of the contribution of NKG2D to the NK cell-mediated killing of infected p24^pos^/CD4^neg^ cell blasts. In line with the phenotypic profiles described and with our previously published results obtained with the *in vitro* infection [17], the masking of NKG2D induced a substantial reduction of NK cell cytolysis of p24^pos^ cell blasts in all patients analyzed (p<0.0001) (Figure 7C). Moreover, we found a significant direct correlation between the expression of ULB-2 on p24^pos^/CD4^neg^ cell blasts and the NK cell-mediated lysis of autologous HIV-1 endogenously infected T cell blasts (Figure 7D). Consistent with results previously published using an *in vitro* experimental system, our data indicate that the pathway activated by interactions of NKG2D with its ULBP ligands (in particular ULBP-2) plays an important role in the NK cell killing of endogenously HIV-1 infected CD4+ T cell blasts [17].

We did not find any functional contribution mediated by the activating NK cell co-receptors 2B4 and NTB-A to lysis of autologous and endogenously HIV-1 infected CD4+ T cell blasts in line with the down-modulation of their cellular ligands, CD48 and NTB-A, on p24^pos^ CD4+ T cell blasts (p = 0.0051 for CD48; p = 0.0049 for NTBA) (Figure S5).

## Discussion

In the present study we demonstrate the ability of NK cells from HIV-1 infected viremic patients to kill endogenously HIV-1-infected autologous CD4+ T cell blasts derived from viremic patients. We show that, subsequent to the selective down-modulation of MHC-I molecules, infected p24^pos^ blasts become partially susceptible to lysis by rIL-2 activated NK cells, while p24^neg^ blasts are spared from killing. This NK cell-mediated killing occurs mainly through the NKG2D activation pathway. However, decreased NK cell expressions of NCRs contribute to the low level of NK cell cytolytic activity. In addition, the unusually high frequency of dysfunctional CD56^neg^ NK cell subsets among HIV-1 infected viremic patients was shown to strongly correlate with the low degree of NK cell cytolytic responses against infected p24^pos^ cell blasts.

The ability of NK cells to kill autologous HIV-1 infected target cells mainly through the NKG2D pathway has been previously demonstrated *in vitro* with exogenously infected CD4+ T cell blasts from healthy donors [15],[17]. In contrast, we examined NK cells from HIV-1 viremic individuals and autologous target cells that were expanded from endogenously infected CD4+ T cells. Given the reported abnormalities in phenotype and functions of NK cells from HIV-infected viremic individuals [26],[27],[28], the present study was designed to determine the function of NK cells in killing HIV-1 infected target cells under conditions that more closely mimic the *in vivo* situation in HIV-infected individuals. Our experimental system relied on rIL-2 activated NK cells instead of freshly purified NK cells. We previously reported that prolonged activation with rIL-2 did not restore phenotype and function of highly dysfunctional NK cells from HIV-1 infected viremic individuals. Only CD56 expression was recovered upon activation with rIL-2 after 3 weeks of culture. Despite the reversion of the CD56^neg^ to a CD56^pos^ phenotype, rIL-2 stimulated CD56^neg^-derived NK cell populations still expressed very high percentages of iNKRs and extremely low levels of NCRs, similar to results from experiments performed with freshly purified CD56^neg^ NK cell subsets. Even after 21 days of activation with rIL-2 the cytolytic potential of these highly dysfunctional NK cells from HIV-1 infected viremic patients did not improve and remained significantly lower compared to that of NK cells from uninfected individuals[7],[8],[11]. Given the fact that stimulation with rIL-2 does not substantially reverse the pathologic characteristics of freshly purified NK cells from HIV-1 infected viremic donors, we designed an experimental system using rIL-2 activated NK cells. This yielded NK cells as effector cells against autologous and endogenously HIV-1 infected CD4+ T blasts at the day of their maximal expansion.

Circulating CD4+ T cells from HIV-infected individuals harbor very low frequencies of replication-competent virus [12],[13]. This has made it very difficult to adequately characterize endogenously infected CD4+ T cells from HIV-infected individuals. Several studies showed that activation *in vitro* with several and multiple stimuli enhanced viral replication in CD4+ T cells from HIV-1 infected patients. The *in vitro* induced replication of HIV-1 was measured by the release of p24 HIV-1 core antigen in culture-supernatant [18],[19],[20],[21]. Using a similar approach for cell activation, we stimulated freshly purified CD4+ T cells from HIV-1 infected viremic patients in order to expand and isolate these endogenously infected T cell blasts detected by intracellular p24 staining. Activation with PHA plus rIL2 (with or without rIL-7) was the most effective in expanding a population of endogenously infected CD4+ T cell blasts. The peak maximal expansion of HIV-1 productively infected blasts was reached, on average, 12 days after activation and the rate of expansion of p24^pos^ cell blasts differed among samples from the 30 HIV-1 infected viremic patients analyzed in the present study. The reasons for such heterogeneous results are unclear and many variables such as cellular or soluble suppressive factors, different numbers of circulating latently HIV-1 infected cells, different viral strains, culture conditions or other factors could contribute to this variability. Further investigations are needed to extensively characterize the kinetics of HIV-1 in endogenously infected cells and the replication cycle of these p24^pos^/CD4^neg^ cell blasts. Our aim in the present study was restricted to an examination of the interactions between endogenously HIV-1 infected CD4+ T cell blasts and autologous NK cells from HIV-1 infected viremic patients.

In the preparation of target cells, we separated infected from uninfected cells on the basis of the lack of expression of CD4 together with intracellular expression of p24 on certain cells (infected) and the expression of CD4 and lack of intracellular expression of p24 on other cells (uninfected). It has been reported that HIV-1 is able to down-modulate the expression of CD4 on T cell surfaces, a phenomenon induced either by Nef, which enhances the internalization and degradation of CD4, or by Vpu and Env, which interfere with the transport of newly synthesized CD4 to cell surface [24],[25]. Even though the physiological relevance of CD4 down-regulation is not fully understood, the absence of CD4 on cell surfaces represents another marker of cells productively infected with HIV-1. We confirmed that endogenously infected CD4+ T cell blasts harboring replication-competent virus down-modulate CD4 expression and, on the basis of surface levels of this molecule, we were able to separate infected p24^pos^ from uninfected p24^neg^ T cell blasts.

In line with results previously reported with HIV-1 infection *in vitro* [15], endogenously HIV-1 infected CD4+ T cell blasts selectively down-modulated HLA-A and –B alleles while the expression of HLA-C and HLA-E molecules was conserved. The selective down-regulation of these MHC-I molecules should render p24^pos^/CD4^neg^ cell blasts susceptible to NK cell-mediated killing. In fact, although the surface levels of HLA-C and -E may still protect infected cell blasts from the cytolysis exerted by autologous NK cells expressing iNKRs specific for these conserved alleles of MHC-I [14],[29], this is not the case for NK cells that express iNKRs specific for HLA-A and –B [15]. We show that the degree of NK cell-mediated lysis of p24^pos^/CD4^neg^ blasts was significantly higher compared with that of p24^neg^/CD4^pos^ blasts. Moreover, masking experiments highlighted the important role of the selective down-modulation of HLA-I molecules, because only the complete blocking of all MHC-I alleles rendered infected p24^pos^ and uninfected p24^neg^ cell blasts equally susceptible to NK cell-mediated lysis.

Other studies reported that conserved or even up-regulated levels of HLA-E on HIV-1 infected cells are able to inhibit NK cell-mediated cytolysis of HIV-1 infected cells through binding to its specific inhibitory receptor NKG2A [14],[30]. In our study, we used two different mAbs (3D12 and 4D12) in order to detect the surface levels of HLA-E. Despite the fact that there was some variability among different donors, we detected no significant differences in the high levels of HLA-E expression between HIV-1 infected and uninfected CD4+ T cell blasts from HIV-1 infected viremic patients. Moreover, given that the frequency of the NKG2A^pos^ NK cell subset is greatly decreased in chronic HIV-1 infected viremic patients compared to that of healthy donors [7],[31], it is unlikely that the interaction between HLA-E and NKG2A can explain the decreased NK cell mediated killing of HIV-1 infected blasts. In fact, our masking experiments demonstrated that the complete blocking of NKG2A did not increase NK cell cytolysis of autologous p24^pos^ blasts (data not shown). The reason for the discrepancy in the role of NKG2A/HLA-E interactions between these previous studies and our data may be the fact that the effector cells used in those previous studies were heterologous NK cell lines expressing high levels of NKG2A against HLA-E transfected target cell lines.

As mentioned above, the engagement of activating NK cell receptors should be able to trigger the cytolytic activity in NK cells expressing iNKRs specific for HLA-A and -B and lacking iNKRs for HLA-C and-E. In this regard, NK cell-mediated killing of infected p24^pos^/CD4^neg^ cell blasts was found to be mainly NKG2D-dependent. These results are in line with the highly conserved expression of NKG2D on NK cells from HIV-1 infected viremic individuals and with the relatively high percentages of p24^pos^ blasts expressing NKG2D ligands. The direct effect of HIV-1 on the positive or negative modulation of NKG2D ligands on the surfaces of primary CD4+ T cells infected *in vitro* with HIV-1 is controversial [17],[32]. We show that masking the binding of NKG2D to its ligands clearly resulted in a marked reduction of the NK cell-mediated lysis of infected blasts. These results suggest that the NKG2D ligands, expressed at high levels in p24^pos^/CD4^neg^ blasts, play an important role in NK cell-mediated killing of autologous infected cells. Several groups previously reported that HIV-1 viremia affects several functions of NK cells and dramatically influences their phenotype [26],[27],[28]. Interestingly, the surface expression and activation pathway of NKG2D are among the few NK cell characteristics spared from the deleterious effects of HIV-1 infection. In order to understand better how HIV-1 affects NK cell cytolytic responses, it would be important for future investigations to address the molecular mechanism(s) underlying the resistance of NKG2D, compared to other important activating and inhibitory NK receptor pathways, to the dysfunction associated with HIV viremia.

Although defective in HLA-A and –B expression, p24^pos^/CD4^neg^ blasts remain still poorly sensitive to killing exerted by autologous NK cells. This is partly the result of inhibitory interactions between iNKRs and conserved HLA-C molecules, as demonstrated by the relatively low levels of killing of both infected and uninfected autologous blasts even in the presence of anti-MHC-I mAbs which completely block the interactions between MHC-I molecules and iNKRs. The relatively low degree of NK cell cytolytic activity against endogenously HIV-1 infected CD4+ T cell blasts might be secondary to aberrancies in NK cell triggering through important activating receptors other than NKG2D. This concept is further supported by the finding that NK cells from HIV-1 infected viremic patients were markedly impaired, compared to that from healthy donors, in their ability to kill highly susceptible target cells such as K562 and 221 tumor cell lines that do not express MHC-I molecules. Moreover, if we compare these experimental data with our previously reported results obtained with HIV-1 infection *in vitro* [17], the degree of killing of autologous, exogenously HIV-1 infected CD4+ cell blasts by NK cells from healthy donors appears to be markedly higher compared to killing by of highly dysfunctional NK cells obtained from HIV-1 infected viremic individuals. In this context, the low levels of NKp46 and NKp30 on NK cells from HIV-infected individuals significantly correlated with NK cell-mediated killing of MHC-I^neg^ K562 and 221 cell lines (data not shown) and of endogenously HIV-1 infected autologous CD4+ T cell blasts. These data suggest that the negative effect of HIV-1 viremia on NKp46 and NKp30 expression interfere with the NK cell lysis of endogenously HIV-1 infected autologous CD4+ T cell blasts. HIV-1 infected autologous CD4+ T cell blasts. Our finding of the negative contribution of NCRs in the killing of endogenously infected targets in HIV-infected viremic individuals differs from the findings of a previous study in which we described that NKG2D was important in NK lysis of infected targets, but that NCRs played no demonstrable role [17]. This discrepancy may result from the fact that the study in question used NK cells from normal individuals and target cells that were infected *in vitro* with several viral strains, whereas the present study employed NK cells from HIV-infected viremic individuals and endogenously infected target cells.

It is well known that HIV-1 viremia induces a CD4+ T cell depletion that leads to immunodeficiency and correlates with disease progression. However, it has also been reported that the majority of CD4+ T cells dying during the infection are not productively infected with HIV-1[33]. One possible explanation is that these uninfected CD4+ T cells are eliminated through a mechanism not directly linked to viral replication. It has been demonstrated both *in vitro* [17] and *ex vivo* (Figure 5B) that HIV-1 replication can modulate the expression of ligands for NKp46, NKp30 and NKp44 on uninfected p24^neg^/CD4^pos^ T cell blasts. In particular, an highly conserved motif of HIV-1 gp41 envelope protein can induce the expression of NKp44 ligand on uninfected CD4+ T cell blasts and render these cells susceptible to NK cell-mediated killing via NKp44 activation pathway[34]. A recent report showed that is possible to prevent the expression of NKp44 ligand on CD4+ T cells, thus providing new insight for both preventive and therapeutic HIV-1 vaccine strategies[35].

In conclusion, the present study shows that NK cells from HIV-1 infected viremic patients display a variable although generally low ability to lyse endogenously HIV-1 infected autologous CD4+ T cell blasts derived from peripheral blood. The selective down-modulation of HLA-A and -B molecules makes p24^pos^/CD4^neg^ cell blasts susceptible, at least in part, to autologous NK cell-mediated lysis mainly through the NKG2D activation pathway. Several other factors including the decreased NK cell expression of NCRs, low levels of NCR-specific ligands on p24^pos^ CD4+ T cell blasts and the high frequency of the dysfunctional CD56^neg^ NK cell subset also contribute to the low levels of NK cell-mediated killing of HIV-1 endogenously infected autologous CD4+ T cell blasts. In fact, the defective killing through the NCR activation pathways and the presence at very high levels of a markedly anergic CD56^neg^ NK cell population substantially impair the ability of NK cell to kill endogenously HIV-1 infected autologous CD4+ T cell blasts. Understanding the mechanisms by which HIV-1 is able to negatively modulate the expression and function of NCRs on NK cell and of their ligands on HIV-1 infected CD4+ T cells will certainly give us new insights for improving the NK cell-mediated lysis of infected cells and for enforcing the innate immune control of HIV-1 infection.

## Materials and Methods

### Study Subjects

Thirty HIV-1 infected viremic individuals were studied. The median CD4+ T cell count was 373 cell per ml (SD = ±193) and the median viremia was 32,677 HIV-1 RNA copies (SD = ±80,734) per ml of plasma as detected by an ultrasensitive branched DNA (bDNA) assay (Chiron) with a lower limit of detection of 50 copies per ml. Patients were either naïve to antiretroviral therpay (ART) or had formerly been receiving ART, but were not receiving therapy at the time of the study. Leukapheresis was conducted in accordance with protocols approved by the Institutional Review Boards (IRBs) of the University of Toronto, Ontario, Canada and the National Institute of Allergy and Infectious Diseases (NIAID), National Institutes of Health (NIH), Bethesda, Maryland, USA. Each patient signed a consent form that was approved by the above IRBs. As negative controls, cells from 30 healthy donors seronegative for HIV-1 were obtained by apheresis generously provided by the Transfusion Medicine Department of the Mark O. Hatfied Clinical Research Center of the NIH as a part of IRB approved clinical studies.

### Isolation and culture of CD4+ T cells and NK cells

PBMCs were obtained from leukapheresis packs by Ficoll-Hypaque density gradient centrigugation (LSM, MP Biomedicals). CD4+ T cells and NK cells were freshly isolated by negative selection (Stem Cell Technologies) according to the protocol provided by the manufacturer. The purity of CD3+/CD4+ T cells was ≥97%. Purified NK cells contained ≤ 3% contamination with other PBMC subsets, as determined by expression of CD3, TCR-a/b, TCR-g/d, CD19 or CD14.

In order to expand CD4+ T cell blasts productively and endogenously infected with HIV-1, we activated freshly purified CD4+ T cells (2×10^6^/ml) with different stimuli, as shown in Figure S1. Briefly, cells were cultured with RPMI medium 1640 supplemented with antibiotics (Gibco) and FCS (HyClone) as previously described[7] and stimulated with phytohemoagglutinin (PHA) (Sigma-Aldrich) at 3 µg/ml for 24 hours plus recombinant IL-2 (rIL-2) (Roche) at 50 IU/ml with or without recombinant IL-7 (rIL-7) (R&D Systems) at 10ng/ml for 21 days. We also activated freshly purified CD4+ T cells with rIL-7 with or without rIL-2 or with soluble anti-CD28 mAbs at 5 µg/ml on tissue culture plates coated with anti-CD3 mAbs at 10 µg/ml (BD-Pharmingen) for 21 days in the presence of rIL-2. Freshly purified NK cells were activated in vitro for 12 days with rIL-2 at 200 IU/ml at 2*10^6^/ml.

### mAbs

The following panel of anti-human monoclonal antibodies (mAbs) were used in this study: mAbs 289 (IgG2a anti-CD3), C218 and A6-220 (IgG1and IgM anti-CD56, respectively), KD1 (IgG2a anti-CD16), AZZ20 and F252 (IgG1 and IgM anti-NKp30, respectively), BAB281 and KL247 (IgG1 and IgM anti-NKp46, respectively), Z231 and KS38 (IgG1 and IgM anti-NKp44, respectively), ON72 and Bat221 (IgG1 anti-NKG2D), MA127 and ON56 (IgG1 and IgG2b anti-NTBA, respectively), pp35 and Co54 (IgG1 and IgM anti-2B4, respectively), Ma152 and CER1 (IgG1 and IgM anti-NKp80, respectively), KRA236 and F5 (IgG1 and IgM anti-DNAM-1, respectively), L14 (IgG2a anti-Nectin 2), L95 (IgG1 anti-poliovirus receptor), Z270 (IgG1 anti-NKG2A), Y9 (IgM anti-CD94), EB6 (IgG1 anti-p58.1/KIR2DL1), Gl183 (IgG1 anti p58.2/KIR2DL2), Z276 (IgG1 anti-p70/KIR3DL1), F278 (IgG1 anti-LIR-1ILT2) and A6.136 (anti-MHC class I molecules, IgM). FITC-, PE- or APC-labeled anti-CD3, anti-CD4, anti-CD8, anti-TCRα/β, anti-TCRγ/δ, anti-CD14, anti-CD19, anti-CD56, anti-MICA/B and anti-CD48 mAbs were purchased from BD Biosciences. Soluble fusion proteins for NKp30, NKp46, NKp44 and NKG2D with the Fc portion of human IgG and anti-human ULBP-1,-2 and -3 mAbs were purchased from R&D Systems. PE-labeled anti-human Fc fragment mAb was purchased from Jackson ImmunoResearch Laboratories. FITC- and PE- anti human anti-HIV-1 p24 mAb (clone KC57) used for intracellular flow cytometry staining was purchased from Coulter Clone. PE-labeled anti-HLA-A mAbs were purchased from Lab Vision Corporation. Anti-human HLA-C mAb (clone L31) was kindly provided by Dr. Patrizio Giacomini (Regina Elena Cancer Institute, Rome, Italy) and used in flow cytometry as previously described[36]. Anti-human HLA-Bw4 (clone 116.5.28) and HLA-BW6 (clone 126.39) mAbs were kindly provided by Dr. Keith Gelsthorpe (National Blood Transfusion Service, Sheffield, UK). Anti-human HLA-E mAbs (clones 3D12 and 4D12) were kindly provided by Dr. Dan Gerarthy (Fred Hutchinson Cancer Research, Seattle, WA, USA).

### Flow cytometry

For one-, two- or three-color cytofluorimetric analysis (FACS Calibur, BD), cells were stained with the appropriate FITC-, PE- or APC-labeled mAbs. For indirect staining, cells were stained with appropriate unlabeled mAbs followed by FITC- or PE-conjugated isotype-specific goat anti-mouse second reagent (Southern Biotechnology Associates). Second appropriate anti-isotypic mAbs stained with FITC and/or PE and/or APC were used as negative controls. For intracellular staining, samples were fixed and permeabilized by cytofix/cytoperm solution and washed with perm-wash solution 1X (BD-Pharmigen) according to the protocol provided from the manufacturer. The data were analyzed using FlowJo software (Tree Star Inc.).

### Detection, proliferation and fluorescence microscopy of endogenously HIV-1 infected CD4+ T cell blasts

The percentages of HIV-1 infected CD4+ T cell blasts were detected by intracellular flow cytometry with an anti-p24 core virus antigen mAb.

Cells undergo cell cycling were evaluated by detecting the intra-nuclear expression of Ki67 (BD-Pharmigen).

CD4-derived T cell blast proliferation was detected by ^3^[H]thymidine uptake assay (16 hours). Cellular proliferation was also evaluated by dilution of the vital dye CFSE (Molecular Probes) according to the supplier's instructions.

After 10–12 days of activation with PHA and rIL-2, unfractionated CD4+ T cell blasts were permeabilized by cytofix/cytoperm solution and stained with an PE-labeled anti-p24 HIV-1 core antigen mAb (Coulter Clone**)** followed by a biotin conjugated mouse anti R-PE mAb (BD). Infected p24^pos^ CD4+ T cell blasts were detected by a Pacific Blue fluorescent-dye conjugate of streptavidin (Molecular probes) according to the supplier's instructions. Fluorescent cells were then washed in PBS, suspended in medium, and sealed on the slides with cover slips. Images were collected on a Leica TCS-NT/SP confocal microscope (Leica) using a 63x oil immersion objective NA 1.32. Pacific Blue was excited using an Argon laser at 364 nm. DIC (differential interference contrast) images were collected simultaneously with the fluorescence images using the transmitted light detector. Images were processed using Leica TCS-NT/SP software (version 1.6.587), Imaris 3.3.2 (Bitplane AG), and Adobe Photoshop 7.0 (Adobe systems).

### Cytolytic activity

In line with the timeframe of maximal expansion of p24^pos^/CD4^neg^ blasts, after 12 days of stimulation we removed by negative selection all contaminant cells from CD4+ T cell blast cultures (MACS, Milteny Biotec). As a result, we obtained a highly purified population of CD4+ T cell-derived blasts containing ≤5% contamination of other lymphocyte subsets (TCRg/d+, CD8+, CD56+, CD16+ and CD19+ cells). HIV-1 infected CD4+ T cell blasts were then separated from uninfected blasts on the basis of CD4 surface expression through magnetic microbeads conjugated with an anti-human CD4 mAb (MACS, Milteny Biotec), according to the protocol provided by the manufacturer. The purities of fractions of uninfected CD4^pos^ and infected CD4^neg^ blasts , as assessed by intracellular staining with HIV-1 p24 core antigens, was ≥97% and ≥70%, respectively. p24^neg^/CD4^pos^ and p24^pos^/CD4^neg^ cell blasts were then used as target cells against autologous rIL-2 activated NK cells in a 4-hour ^51^Cr release assay as described previously[37]. Saturating concentration (10 µg/ml) of specific mAbs blocking NK cell receptors or MHC-I molecules were added for the masking experiments. The NK cell:T cell blast ratio was 10∶1 (Figure S1).

Polyclonal NK cells were also tested in a 4-hour ^51^Cr release assay against MHC-I^neg^ erythroleukemia K562 and MHC-I^neg^ B-EBV cell line 721.221 (thereafter termed 221). E/T ratios are indicated in the figures.

^51^Cr release cytolytic assay were performed on cells from 15 HIV-1 infected viremic patients.

### Statistical Analysis

Immune response distributions between healthy donors and HIV-1 infected viremic patients were compared using the Mann-Whitney test. The phenotypic and functional differences between p24^pos^ and p24^neg^ blasts from HIV-1 infected individuals were evaluated using the Wilcoxon signed ranks test. The functional differences between NK cell-mediated baseline lysis and lysis in masking experiments were evaluated using the Wilcoxon signed ranks test. All p-values are 2-sided and unadjusted. All statistical associations between different immune parameters were determined by the Spearman rank test for correlation. To estimate the time of maximal infection, the mean outcome of infected p24^pos^ over time was modeled as a polynomial function of time and estimated using least squares. The maximum of this function was then determined and a bootstrap procedure was used to provide a confidence interval ≥95% for the maximum.

## Supporting Information

Figure S1 Methodology. p24^pos^ blasts were expanded from total PBMCs obtained from HIV-1 infected viremic patients and used as targets for autologous rIL-2 activated NK cells
(0.05 MB PDF)

Figure S2 Expansion *ex vivo* of HIV-1 infected CD4+ T cell-derived blasts by using different stimuli. Percentages of p24^pos^ blasts (open squares) expanded at day 12 after activation with rIL-2 or rIL7 alone, with PHA plus rIL-7 or rIL-2±rIL-7 and with anti-CD3 plus anti CD28 mAbs from a representative HIV-1 infected viremic patient.
(0.04 MB PDF)

Figure S3 Sorting of HIV-1 infected and uninfected CD4+ T cell-derived blasts. Representative example of separation of p24^pos^ from p24^neg^ blasts through magnetic microbeads conjugated with an anti-CD4 mAb. Purities of sorted uninfected CD4^pos^ and infected CD4^neg^ T cell blast fractions were assessed by intracellular staining with HIV-1 p24 core antigens in a double color flow cytometric analysis.
(0.04 MB PDF)

Figure S4 Cytolytic Activity of NK cells against HLA-I^neg^ tumor target cell lines. Spontaneous killing of K562 (A) and 221 (B) tumor cell lines by rIL-2 activated NK cells-. Data are presented as the average of experiments conducted on 15 healthy donors (black squares) and 15 HIV-1 infected viremic patients (red diamonds).
(0.01 MB PDF)

Figure S5 NK cell-mediated killing of autologous HIV-1 infected CD4+ T cell-derived blasts: role of 2B4 and NTBA and expression of their ligands on cell targets. (A-B) Surface expression of CD48 and NTBA in p24^neg^ (upper left quadrants of dot plot graphs and green lines in histogram graphs) and p24^pos^ (upper right quadrants of dot plot graphs and red lines in histogram graphs) blasts derived from a representative HIV-1 infected viremic patient. Data are indicated as percentage of expression (A) and as MFI (b). (C) Cytolysis (in triplicate ±SD) of autologous p24^neg^/CD4^pos^ blasts exerted by rIL-2 activated NK cells purified from a representative HIV-1 infected viremic patient. Cells were incubated either in the absence (baseline lysis) or in the presence of specific mAbs masking 2B4 and NTBA. We used an anti-human CD56 IgM mAb as an isotype control for masking experiments. The NK cell:CD4-derived blast ratio in all experiments was 10∶1.
(0.04 MB PDF)
