# Supplementary figures and images for: Lysis of Endogenously Infected CD4+ T Cell Blasts by rIL-2 Activated Autologous Natural Killer Cells from HIV-Infected Viremic Individuals
Source: PLoS Pathog. 2008 Jul 11;4(7):e1000101. doi: 10.1371/journal.ppat.1000101 (PMC2438610; doi:10.1371/journal.ppat.1000101)

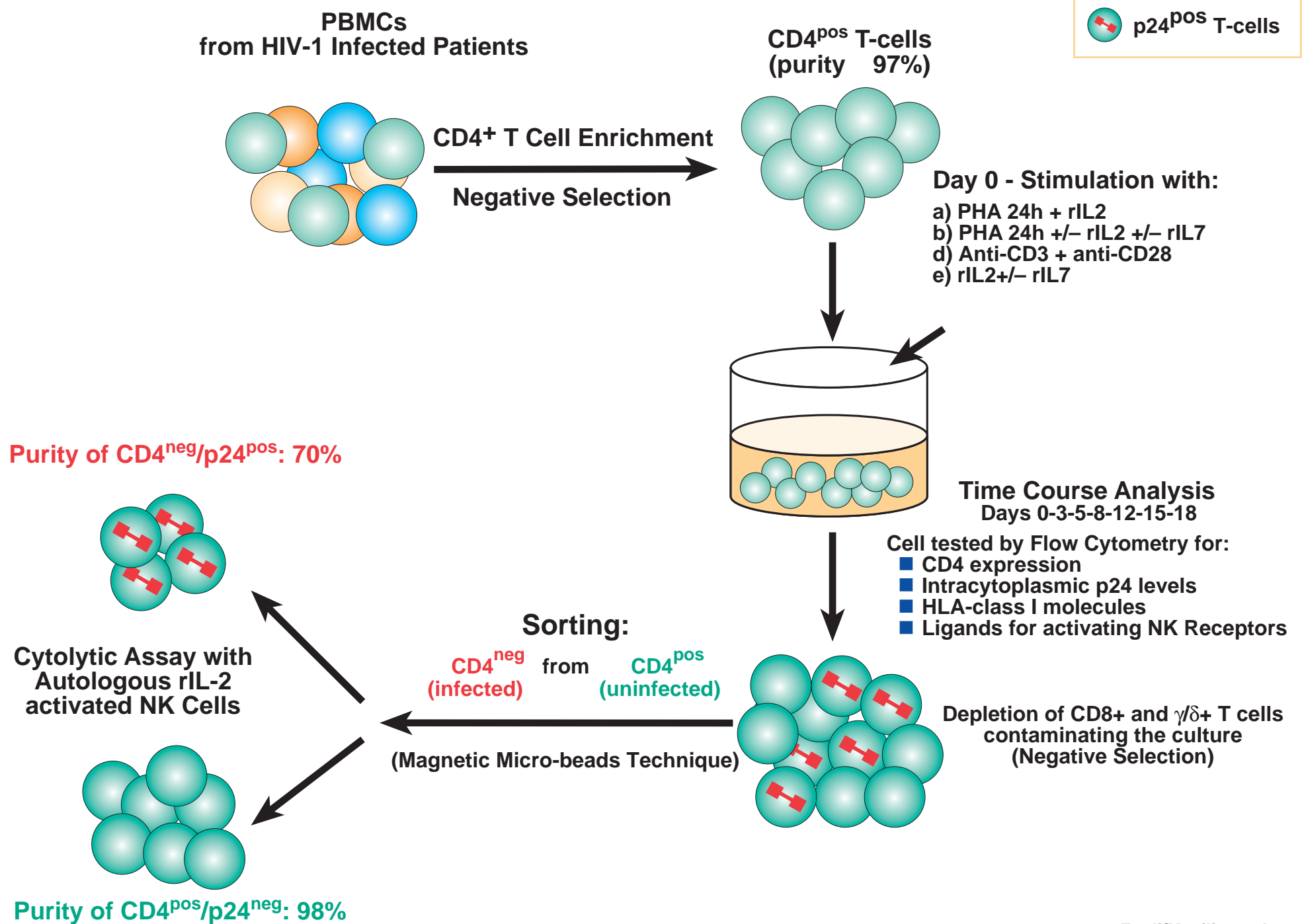

Supplement: Figure S1 — Methodology. p24pos blasts were expanded from total PBMCs obtained from HIV-1 infected viremic patients and used as targets for autologous rIL-2 activated NK cells (0.05 MB PDF) [file ppat.1000101.s001.pdf]

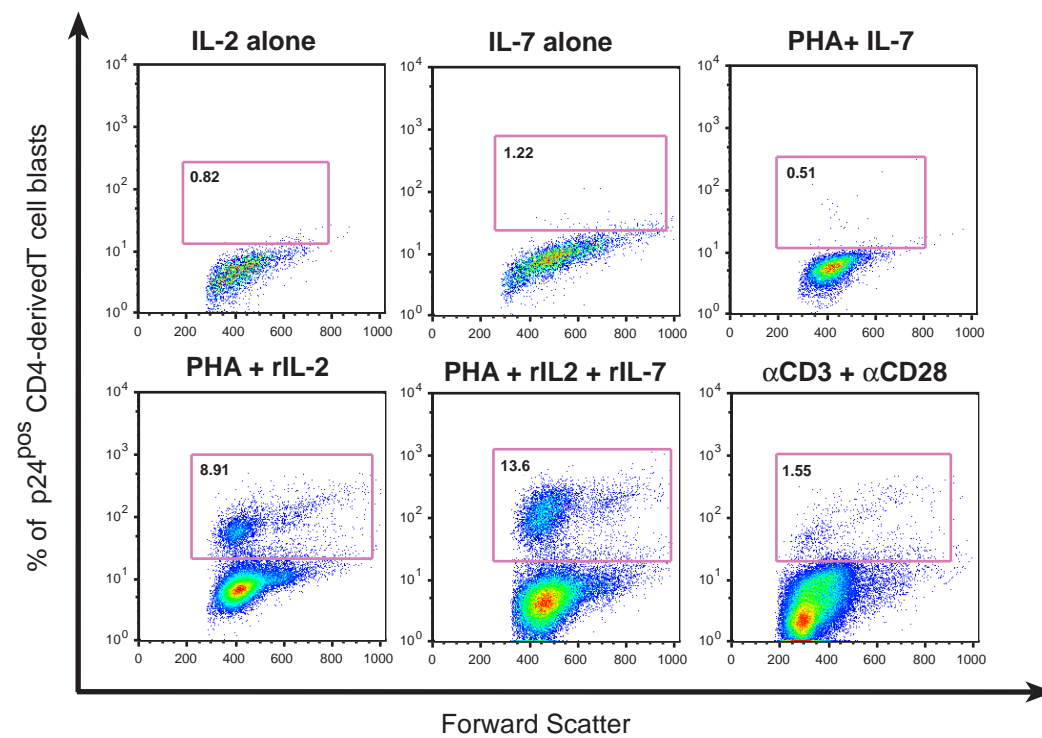

Supplement: Figure S2 — Expansion ex vivo of HIV-1 infected CD4+ T cell-derived blasts by using different stimuli. Percentages of p24pos blasts (open squares) expanded at day 12 after activation with rIL-2 or rIL7 alone, with PHA plus rIL-7 or rIL-2±rIL-7 and with anti-CD3 plus anti CD28 mAbs from a representative HIV-1 infected viremic patient. (0.04 MB PDF) [file ppat.1000101.s002.pdf]

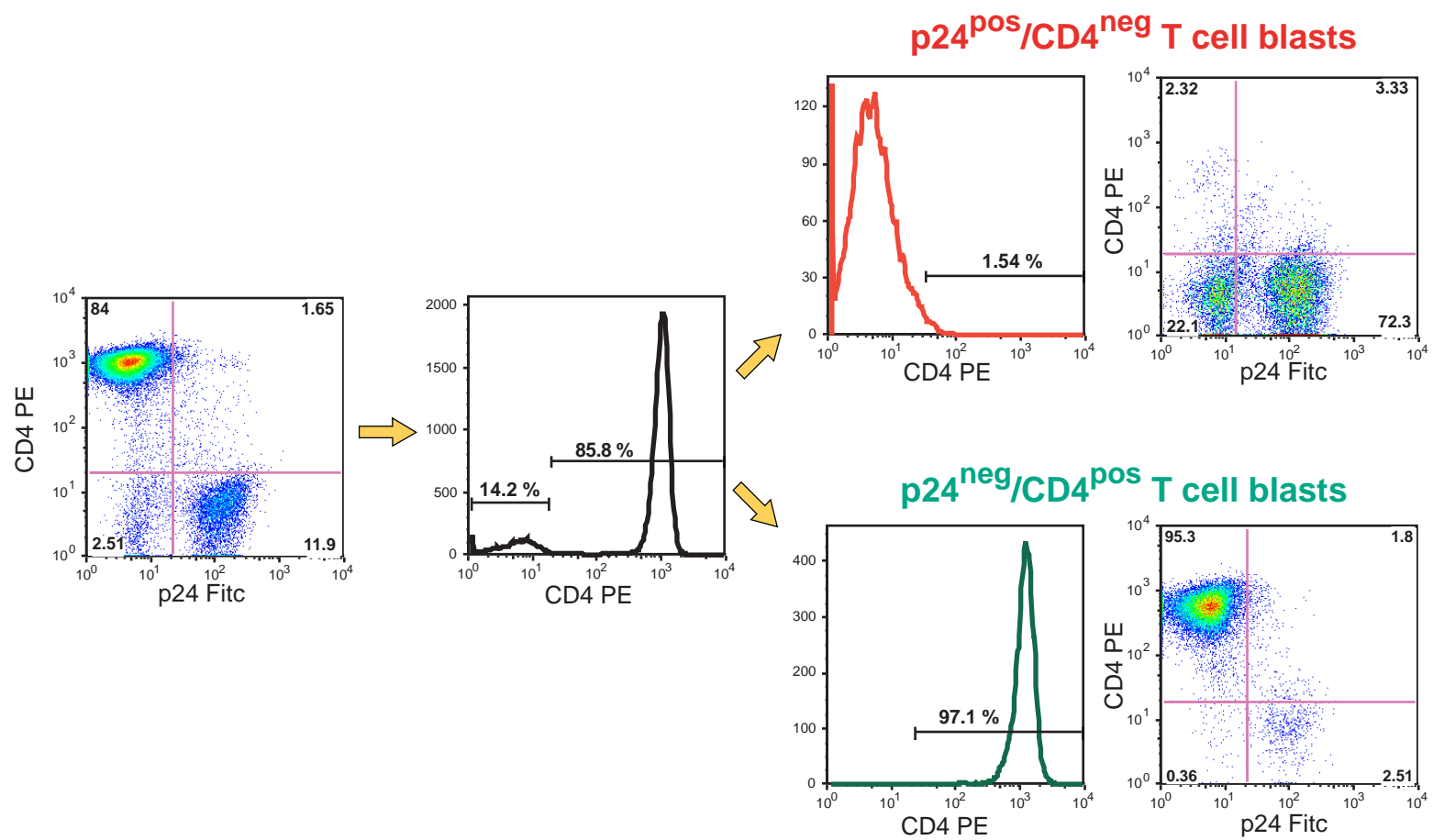

Supplement: Figure S3 — Sorting of HIV-1 infected and uninfected CD4+ T cell-derived blasts. Representative example of separation of p24pos from p24neg blasts through magnetic microbeads conjugated with an anti-CD4 mAb. Purities of sorted uninfected CD4pos and infected CD4neg T cell blast fractions were assessed by intracellular staining with HIV-1 p24 core antigens in a double color flow cytometric analysis. (0.04 MB PDF) [file ppat.1000101.s003.pdf]

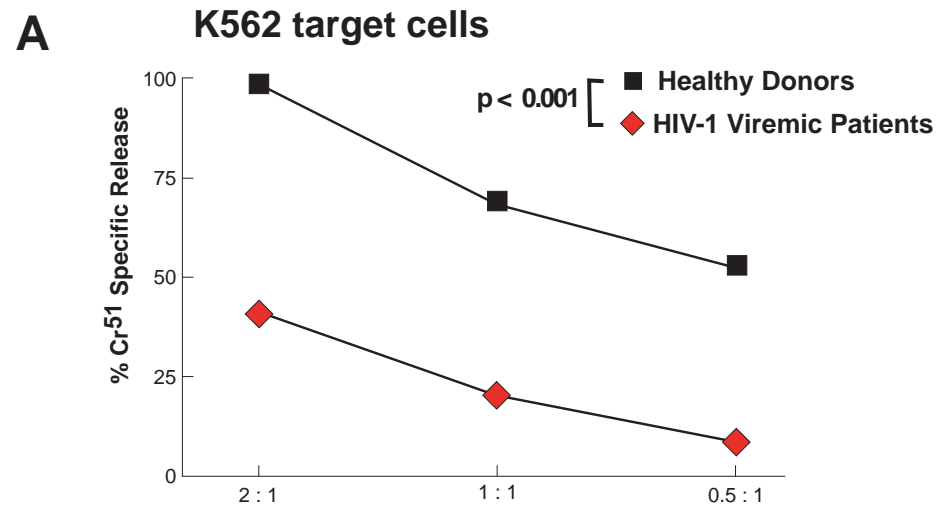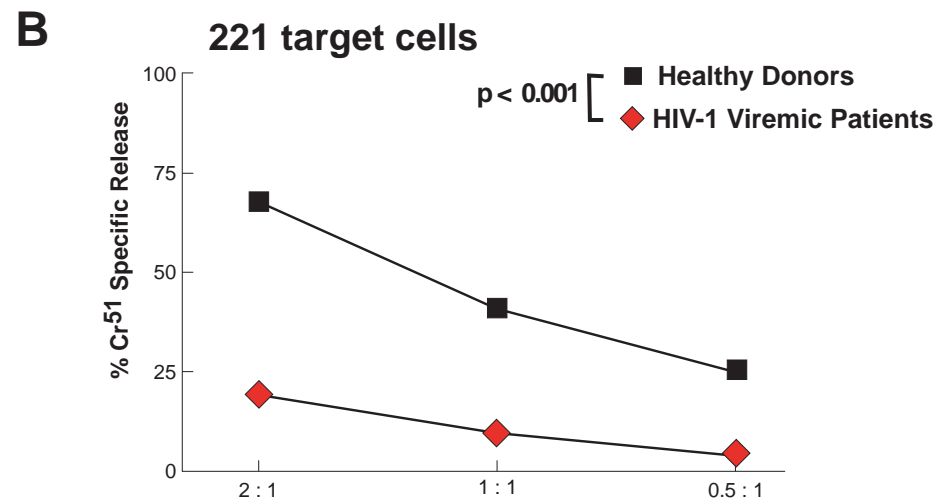

Supplement: Figure S4 — Cytolytic Activity of NK cells against HLA-Ineg tumor target cell lines. Spontaneous killing of K562 (A) and 221 (B) tumor cell lines by rIL-2 activated NK cells-. Data are presented as the average of experiments conducted on 15 healthy donors (black squares) and 15 HIV-1 infected viremic patients (red diamonds). (0.01 MB PDF) [file ppat.1000101.s004.pdf]

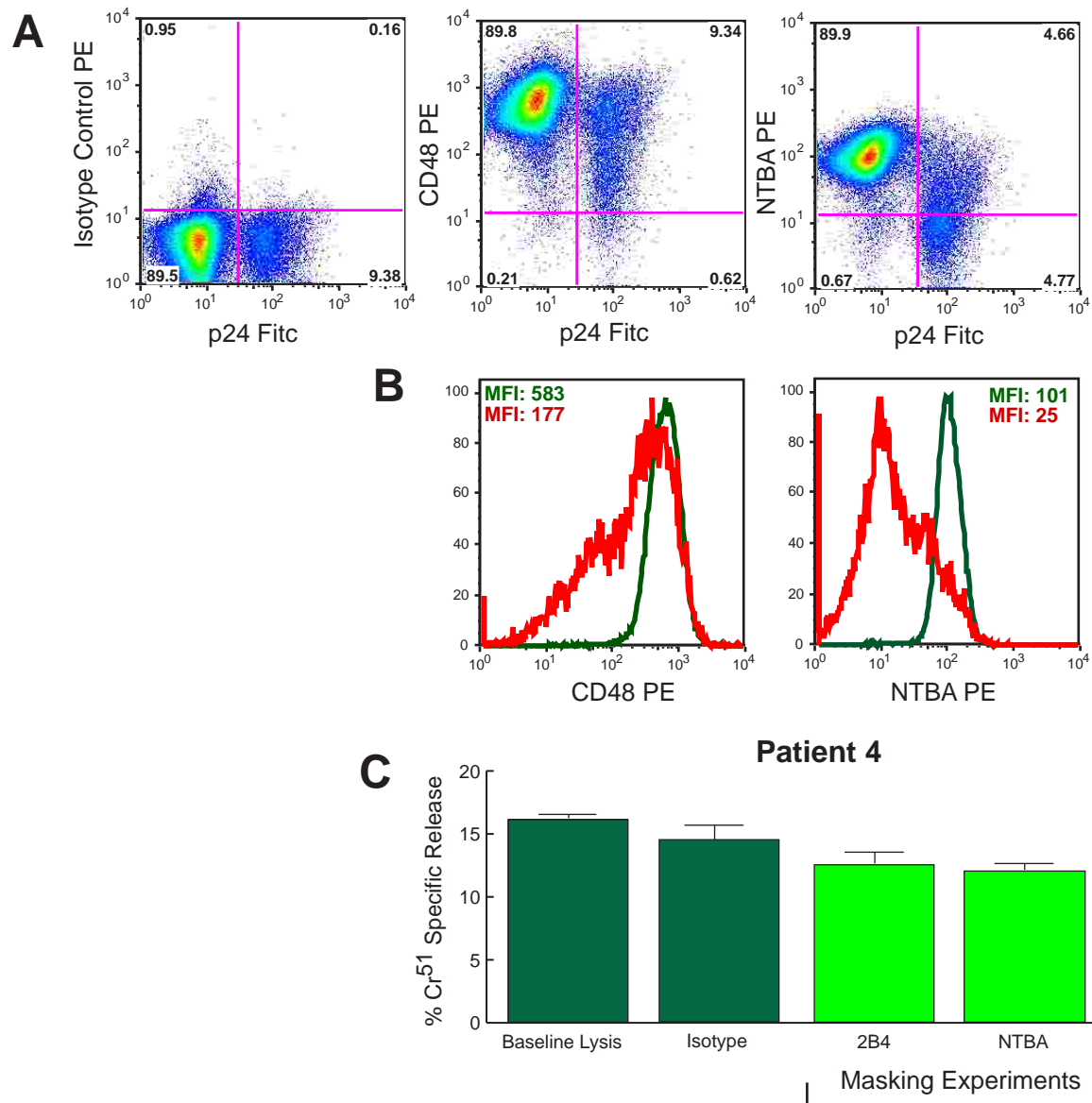

Supplement: Figure S5 — NK cell-mediated killing of autologous HIV-1 infected CD4+ T cell-derived blasts: role of 2B4 and NTBA and expression of their ligands on cell targets. (A-B) Surface expression of CD48 and NTBA in p24neg (upper left quadrants of dot plot graphs and green lines in histogram graphs) and p24pos (upper right quadrants of dot plot graphs and red lines in histogram graphs) blasts derived from a representative HIV-1 infected viremic patient. Data are indicated as percentage of expression (A) and as MFI (b). (C) Cytolysis (in triplicate ±SD) of autologous p24neg/CD4pos blasts exerted by rIL-2 activated NK cells purified from a representative HIV-1 infected viremic patient. Cells were incubated either in the absence (baseline lysis) or in the presence of specific mAbs masking 2B4 and NTBA. We used an anti-human CD56 IgM mAb as an isotype control for masking experiments. The NK cell:CD4-derived blast ratio in all experiments was 10∶1. (0.04 MB PDF) [file ppat.1000101.s005.pdf]
